# Functional outcome and health-related quality of life following ipsilateral femoral and acetabular fractures: a retrospective analysis

**DOI:** 10.1051/sicotj/2021050

**Published:** 2021-10-28

**Authors:** Abdullah Said Hammad, Ramy Ahmed Rashed, Ghada Abu-Sheasha, Ahmed El-Bakoury

**Affiliations:** 1 Associate Professor Orthopaedic Surgery and Traumatology, Elhadra University Hospital, Faculty of Medicine Alexandria Egypt; 2 Clinical Fellow Trauma and Orthopaedics, University Hospitals Plymouth NHS Trust Plymouth PL68DH UK; 3 Associate Professor of Biomedical Informatics and Medical Statistics, Medical Research Institute, Alexandria University Alexandria Egypt; 4 Lecturer of Orthopaedics and Trauma, University of Alexandria Alexandria Egypt; 5 Consultant Orthopaedic Surgeon, University Hospitals Plymouth NHS Trust Plymouth PL68DH UK

**Keywords:** Floating hip, Quality of life, Functional outcome, Ipsilateral femoral and acetabular fractures

## Abstract

*Introduction*: The combination of ipsilateral femoral and acetabular fractures is known in the literature as the “Floating hip injury”. The primary aim of this study is to assess both generic and specific patient-reported outcomes and the factors affecting the quality of life in patients sustaining this injury, while the secondary aim was to assess the injury patterns and the associated complications. *Methods*: A retrospective study including 27 patients according to specific inclusion and exclusion criteria. EQ5D5L and Oxford hip score (OHS) were used. The mean age was 28 years (±10.1 *SD*) and 21 patients (77.8%) were males. The mean follow-up was 7 years (± 3.1 years *SD*). *Results*: Median OHS was 46.5 (IQR: 31.5–48). The median EQ5D score was 0.919 (95% CI: 0.601–1). The mean EQ5D index value was 0.679 ± 0.442 (95% CI: 0.492–0.865). In this young cohort of patients, this drop in the mean EQ5D index value has led to a loss of a mean of 2.2 Quality-adjusted Life Years (QALYs). Through multivariate analysis, we found that the quality of life was mainly affected by the occurrence of end-stage arthritis, the presence of non-recovered traumatic sciatic nerve injury, and the occurrence of infection. *Conclusions*: Our findings show that the quality of life of those patients was significantly affected. These findings can be beneficial in counselling patients sustaining this complex injury and could be helpful in the discussion of the prognosis and in planning postoperative rehabilitation and support.

## Introduction

The combination of an ipsilateral femur and acetabular fracture is uncommon, and it has been estimated that the incidence is 1 in 10,000 [1, 2]. Various case reports have been published to describe the management of this injury [3–12], however, few studies have been published to describe the injury patterns and the associated complications.

Judet et al. [13] defined 10 different types of acetabular fractures, stating that the position of the femoral head at the time of trauma indicates the fracture type. Tile [14] described two acetabular fracture mechanisms according to the direction of force applied: a “dashboard” type injury and a “side blow” injury to the greater trochanter. This classical biomechanical interpretation of acetabular fractures was never proven by laboratory studies. Liebergall et al. [15] reinforced Tile’s observations and formulated a patho-mechanical classification, in which they described that a lateral impact injury to the trochanter would result in a central fracture of the acetabulum associated with a proximal femoral fracture. A dashboard injury would result in a posterior fracture/dislocation of the acetabulum associated with a diaphyseal or distal femoral fracture and/or bony or ligamentous injuries to the knee.

This study aimed primarily to evaluate the health-related quality of life (HRQoL) outcome of patients with ipsilateral femoral and acetabular fractures using combined generic and specific patient-reported outcome measures (PROMs) and secondarily was to assess the injury patterns and the associated complications.

## Materials and methods

After approval from the local ethical committee, we reviewed the medical records for patients who underwent surgical treatment for acetabular fractures between 2008 and 2019 at our tertiary referral university hospital. The inclusion criteria were patients with ipsilateral acetabular and femoral fracture following high energy trauma and had a minimum 1 year follow-up. Exclusion criteria included patients with insufficiency fractures following low energy trauma, and those with open femoral fractures.

A total of 434 patients with surgically treated acetabular fractures were reviewed and 27 patients (6.2%) were found to have an ipsilateral femoral fracture and met our inclusion criteria. Basic demographic data and surgical details were collected.

Records showed that the femoral fractures were fixed initially, followed by acetabular fixation. The patient was positioned in supine using a traction table, and standard surgical incisions were used for either femoral nailing or plating. Subsequently, supine position on a standard operative table was used for anterior acetabular fixation, lateral positioning was used for posterior wall fracture fixation, and the prone position was used for posterior fixation of transverse or T-type acetabular fractures. Both femoral and acetabular fixations were performed in the same surgical setting.

**Figure 1:**
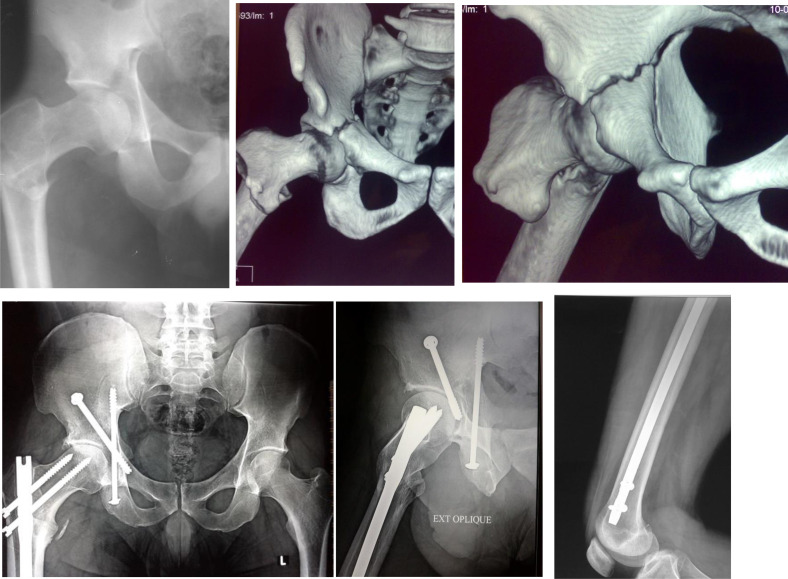
Case example showing ipsilateral acetabular fracture and proximal femoral fracture. Top row: Preoperative X-ray and 3D- reconstruction CT scan. Bottom row: X-rays at 7 years postoperative.

Various surgical strategies were used in the included cohort. One patient (3.7%) was treated with primary total hip replacement (THR). On the acetabular side, four cases (14.9%) were treated with anterior fixation, 10 cases (37%) were treated with posterior fixation, 7 cases (25.9%) were treated with combined anterior and posterior fixation, and 5 cases (18.5%) were treated with percutaneous column screws (Figure 1). On the femoral side, 11 cases (40.7%) were treated with surface fixation and 15 cases (55.6%) were treated with intramedullary fixation.

Of the 27 eligible patients, 24 were available for final analysis. Three patients (11%) were lost to the final follow-up; one patient was deceased following cardiac arrest in 2017, one moved abroad, and one moved out of the area (patient was not contactable). The mean age was 28 years (±10.1 *SD*) and 21 patients (77.8%) were males. The mean period of follow-up was 7 years (±3.1 *SD*).

The telephone version of the EQ5D5L questionnaire was used to obtain scores in telephone interviews in concordance with the EuroQol Group guidebook [16]. The EQ5D5L includes the following domains: mobility, self-care, usual activity including work, pain/discomfort, and anxiety/depression, in addition to a general state of health visual analogue scale (VAS) score. The recorded responses were used to formulate an index value. The index values for the general population in the country where this study took place ranges from −0.93 to 1 [17].

The hip function was assessed by the Oxford hip score (OHS). If the patient sustained injury around the knee, Oxford knee score (OKS) was also obtained.

Preoperative, postoperative, and the most recently available plain radiographs were reviewed to assess of acetabular fracture type, comminution, surgical reduction, signs of avascular necrosis (AVN), heterotopic bone formation, and post-traumatic arthritis. Radiographic assessment of the acetabular and femoral fracture types was done using the AO classification and using the acetabular classification proposed by Liebergall (central and posterior) [15].

### Statistical analysis

The data were analyzed using MedCalc. We used the median and interquartile range (IQR) for measuring central tendency as the data was not normally distributed. Spearman’s correlation was used for measuring the strength of associations while the Mann–Whitney test was used to measure the difference in the outcome variables.

## Results

**Figure 2:**
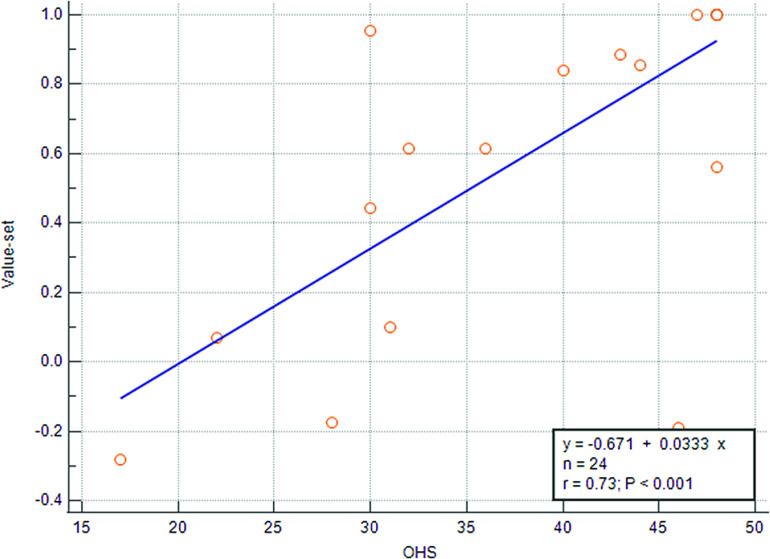
Correlation between Oxford hip score (OHS) and the value set of the EQ5D5L in 24 patients with ipsilateral femur and acetabular fracture.

Table 1 summarizes the clinical outcomes for the included patients at the last follow-up. The median OHS for the included cohort was 46.5 (IQR: 31.5–48). There was a strong positive association between the OHS and the EQ5D5L index value (*ρ* = 0.82, 95% CI: 0.62–0.91, *p* < 0.0001). This is shown in Figure 2. The median OKS was 46 (IQR: 32–48) points. The OKS was used in 12 patients (44.4%). One patient had an associated tibial fracture that was fixed with an intramedullary nail, two patients had associated patellar fractures, four patients had segmental femoral fractures with a distal fragment, four patients had distal femoral fractures, and one patient had a stiff knee because of postoperative infection of femoral intramedullary nail.

**Table 1:** OHS/OKS and EQ-5D value index for the included patients.

	*N*	Minimum	Maximum	Mean	Median	*SD*	RSD	IQR
EQ-5D Value index	24	−0.280	1.000	0.679	0.919	0.4419	0.6509	0.503–1.000
OHS	24	17.000	48.000	40.583	46.500	9.6995	0.2390	31.500–48.000
VAS	24	0.000	100.000	68.375	80.000	29.2646	0.4280	50.000–90.000
OKS	12	26.000	48.000	41.000	46.000	8.7655	0.2138	32.500–48.000

OHS: Oxford Hip Score, OKS: Oxford Knee Score, VAS: Visual analogue scale, SD: standard deviation, RSD: Relative standard deviation, IQR: Interquartile range.

The median EQ-5D index value for the cohort was 0.919 (95% CI: 0.601–1). The mean EQ-5D index value for the included patients was 0.679 ± 0.442 (95% CI: 0.492–0.865).

Bivariate analysis was used to study the factors affecting the final quality of life outcome. This analysis showed that the median EQ-5D index value was lower among patients with postoperative infection, end-stage arthritis, and non-recovered sciatic nerve injury. This effect was statistically significant (*p* = 0.0089, *p* = 0.015 and *p* = 0.0029 respectively) (Table 2). A simple linear regression model was used to quantify the effects of these factors on the EQ5D index value. This showed that end-stage arthritis resulted in a reduction of 0.434 points in the EQ-5D index value (95% CI: −0.86 to −0.005, *p* = 0.047), non-recovered sciatic nerve injury led to a drop of 0.55 points (95% CI: −0.918 to −0.18, *p* = 0.0054), and infection led to a drop of 0.910 points (95% CI: −1.326 to −0.495, *p* = 0.0001).

**Table 2:** Factors affecting the EQ-5D value index at final follow-up.

Domain	Number (%)	Median (IQR)	Test value	*p*-value
Age (years)	24 (100)	26 (21.25, 34.0)	*r* = −0.097	0.65
Follow-up (years)	24 (100)	7 (6, 9)	*r* = −0.15	0.45
Male gender	20 (83.33)	0.919 (0.27, 1)	*U* = 31.00	0.46
	4 (16.67)	0.927 (0.84, 1)		
End-stage arthritis	4 (16.67)	0.563 (0.006, 0.614)	*U* = 15	0.015*
	20 (83.33)	1 (0.84, 1)		
Non-recovered SNI	6 (25)	0.27 (0.07, 0.56)	*U* = 11.50	0.0029*
	18 (75)	1 (0.85, 1)		
Infection	3 (12.5)	−0.174	*U* = 3	0.0089*
	21 (87.5)	1 (0.614, 1)		
Post-dislocation	7 (29.17)	0.885 (0.20, 1)	*U* = 56.5	0.84
	17 (70.83)	0.953 (0.53, 1)		
Polytrauma	11 (45.83)	0.838 (0.16, 1)	*U* = 54.5	0.3
	13 (54.17)	1 (0.60, 1)		

*r*: Spearman correlation coefficient, *U*: Mann–Whitney *U*-test, SNI: Sciatic nerve injury.

The acetabular and femoral fracture patterns are summarized in Tables 3 and 4, respectively.

**Table 3:** Arbeitsgemeinshaft fűr osteosynthesefragen (AO) types of the acetabular and femoral fractures.

AO Acetabulum				
AO Femur	62a	62b	62c	Total *n* (%)
31	4	5	1	10 (37)
32	2	8	2	12 (44.4)
33	0	4	1	5 (18.5)
Total *n* (%)	6 (22.2)	17 (63)	4 (14.8)	27 (100)

**Table 4:** Distribution of fracture patterns according to Liebergall’s mechanism related classification.

	Acetabulum		
Femur	PW intact	PW fracture	Total (%)
Proximal	5*	5	10 (37)
Non-proximal	11	6**	17 (63)
Total *n* (%)	16 (59.3)	11 (40.7)	

* Classic central.

** Classic posterior types according to Liebergall mechanism related classification. PW: Posterior wall.

The complications encountered in the included patients were:

- Infection (one case on femoral side, one case on acetabular side, one case that had infection on both acetabular and femoral sides (12.5%)).
- End-stage arthritis (four cases (16.7%)). One patient underwent THR while one patient is awaiting to have THR. The remaining patients have radiological end-stage arthritis but had no functional limitation to warrant surgical intervention at the final follow-up.
- Non-union of femoral fracture (two cases (8.33%)). One was treated with a circular frame, and the other was a segmental femur fracture with non-union of the distal part, which was treated with exchange nailing.
- Traumatic sciatic nerve injury (eight cases (33.3%)). At 6 months postoperatively, only two patients recovered, leading to a non-recovered sciatic nerve injury incidence of 25% (six cases).
- Heterotopic ossification (five cases (20.8%) Brooker 1, two cases (8.3%) Brooker 2, one case (4.2%) Brooker 3).

## Discussion

The combination of acetabular and/or pelvic injury with a femoral fracture (known as the “Floating hip”) is rare and has been described in a few reports in the literature which are summarized in Table 5 [15, 18–21].

**Table 5:** Studies reporting ipsilateral femoral and acetabular fractures.

	Year	Journal	Number of the patients	Follow-up months (range)	Specific PROMS	Generic PROMS	Complications	Traumatic SNI	Infection	HO
1. Liebergall et al. [18]	1992	Bone and Joint Journal	17	20 (6–35)	NA	NA	100%	60%	20%	20%
2. Müller et al. [19]	1999	Archive Orthopaedic Trauma Surgery	40	N/A	NA	NA	48%	24%	5.8%	47%
3. Liebergall et al. [15]	2002	Injury	20	74 (12–160)	HHS	NA	NA	NA	NA	NA
4. Cannada et al. [20]	2017	Journal of Orthopaedic Trauma	101	11 (3–80)	NA	NA	85%	NA	17%	29%
5. Zamora et al. [21]	2017	Injury	13	Minimum 84	NA	EQ5D	Vascular injury, heterotopic ossification, arthritis, postsurgical neurological injury	NA	NA	7 patients
6. This study	2020		27	84	OHS OKS	EQ5DL	12.5% infection, 29.2% end-stage arthritis, 8.33% femoral nonunion, 33.3% SNI	33.3%	12.5%	0%

There are some limitations to our study. The analysis was retrospective, and the sample size is relatively small, but this can be expected given the rarity of the injury and given that this study reports the experience of a single Level I trauma centre. The small sample size has precluded multiple regression analysis from assessing the strength of the relationship and the importance of each predictor to the outcome.

Strengths of the current study include the analysis of both functional outcome scores (OHS and OKS) in addition to the EQ-5D scores and the factors influencing the overall quality of life, which are important to enable orthopaedic surgeons to discuss the prognosis of these injuries with patients. To our knowledge, this is the largest report which assesses the functional outcome of patients with such rare injury (Table 5). There are a few reports on the functional outcome and quality of life after floating hip injuries. Zamora-Navas et al. [21] have analyzed 25 cases of floating hip injury and compared their outcomes to a control group of 56 patients. However, they only included 13 patients with ipsilateral acetabular and femoral fractures. They have concluded that the quality of life (measured by EQ-5D) was generally worse in the presence of a vascular injury at presentation and the occurrence of heterotopic ossification.

The current study shows similar findings to Zamora Navas et al. [21] report, with the advantage of reporting the results of a bigger cohort of patients using generic and specific outcome measures and how they correlate. We have demonstrated a mean 0.321 units drop in the EQ-5D index value from full health (index value of 1) in patients with floating hip injuries. Assuming that there is no other significant change in their health condition, this drop in the EQ5D has resulted in the loss of approximately 2.2 years (0.321 × 7 years mean follow-up) of Quality-adjusted life years (QALY) over the mean period of follow-up. Since our mean age was 28 years, this drop in QALY can significantly affect their ability to return to work, effects on future earnings, and other aspects of life.

In addition, the current study shows a statistically significant positive correlation between the hip function at the final follow-up (represented by the OHS) and the final EQ-5D index value. This study also shows that the main factors affecting the final EQ-5D index value were the occurrence of end stage arthritis, the presence of non-recovered sciatic nerve injury (SNI) beyond 6 months, and the occurrence of infection.

**Figure 3:**
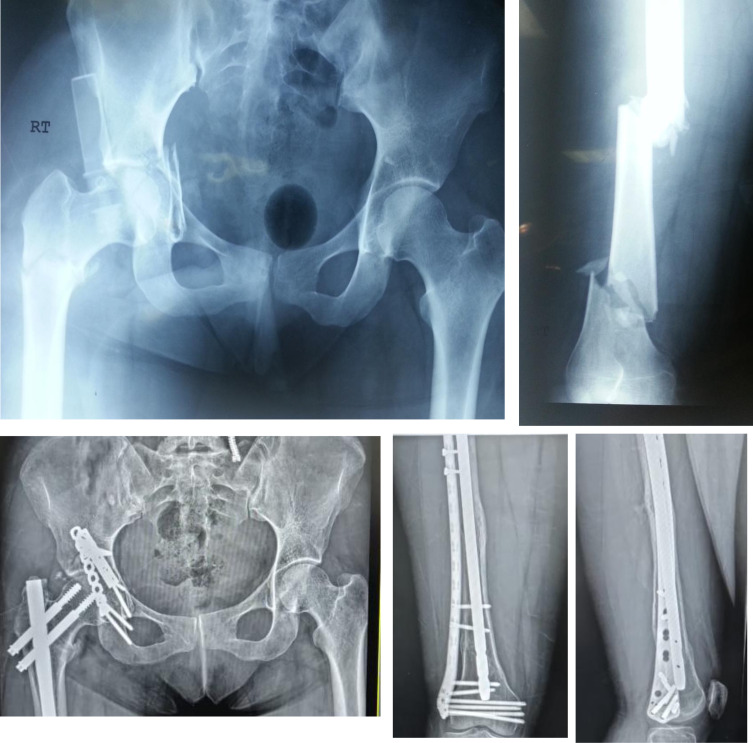
Top row: Preoperative X-rays for an ipsilateral acetabular and segmental femoral fracture, treated with fixation of acetabulum through posterior approach, and femoral nailing followed by plate augmentation for nonunion of midshaft fracture. Bottom row: 6 year follow-up X-rays.

Some studies have related the fracture patterns to the causative mechanism. Liebergall et al. [15] found a strong correlation between the occurrence of a posterior type acetabular fracture and a mid-shaft femoral fracture, as a dashboard type injury causes both. They also found a strong correlation between proximal femoral fractures and a central type acetabular fracture as a lateral trochanteric impaction injury causes both. None of their analyzed patients sustained a distal femoral fracture. The findings in our study were different, as 50% of the patients who sustained a proximal femoral fracture were associated with a posterior-type acetabular fracture. In addition, 12 patients (44.4%) in our cohort had associated distal femoral fracture (either pure distal or a segmental with a distal fragment) and an acetabular fracture (Figure 3) contradicting Liebergall’s theory that energy is dissipated in the distal femur and the residual energy is not sufficient to cause an acetabular fracture. This might reflect the significant high-energy trauma that patients in this cohort have been exposed to, explaining the incidence of associated skeletal injuries (54.6%) and polytrauma (59.1%). Moreover, we believe that awareness of these possible associations is important in the initial assessment of those patients.

Our cohort showed that 33.3% (eight patients) of the cases presented with a traumatic SNI, all affecting the peroneal component of the nerve with a 6-months recovery rate of only 8.3% (two patients). It is very difficult to attribute these results to either the acetabular or the femoral fractures. It is difficult to compare our reported SNI incidence to similar reports in the literature [22, 23] as these reports did not specifically report the incidence of their traumatic SNI after ipsilateral acetabular and femoral fractures, and they have compiled both acetabular and pelvic fractures as a single fracture entity. However, the time of recovery of the sciatic nerve after acetabular fractures and the final functional outcome has been described variably in different series in the literature. Fassler et al. [24] showed that 13 out of 14 patients had satisfactory functional outcomes after 27 months of SNI after an acetabular fracture, but 11 had residual neurological manifestations ranging from paraesthesia to foot drop. The best outcome was found in cases with mild involvement of the peroneal component. Mears et al. [25] reported the results of 16 traumatic SNIs associated with acetabular fractures and concluded that the prognosis was good since 14 of the patients had complete recovery within 6 months of the injury, and only two needed a brace for foot-drop. Epstein [26] reported 38 injuries of the nerve associated with posterior fracture-dislocations of the hip. In 23, the nerves recovered in 3–33 months post-injury, and recovery was poor (partial or none) in 11 patients (29%).

In addition to end-stage arthritis and SNI, we also report a 12.5% incidence of infection, and 8.33% incidence of non-union of the femoral shaft. These results are similar to those reported in the report by Cannada et al. [20], which reports a 17% incidence of infection. They also reported a strong association between the occurrence of avascular necrosis and proximal femoral fractures. This observation could not be assessed in our study due to the small sample size.

The reported incidence of heterotopic ossification with floating hip injuries is 29% [20] and 34% [27] in the literature. In our study, the incidence of heterotopic ossification was 33.3%. Most of these cases were Brookers 1 and 2, which were not clinically significant, hence analysis on the effect of heterotopic ossification EQ-5D could not be assessed.

In conclusion, we believe that the findings of this study should be considered during the assessment, management planning, and counselling of the affected patients. Our findings show that the quality of life of those patients was significantly affected, hence all efforts should be made to provide the appropriate post-surgical rehabilitation and support.

## Funding

The authors declare that no funding has been received for this work.

## Conflict of interest

The authors declare that they have no conflict of interest.

## Ethical approval

This research was conducted retrospectively from data obtained for clinical purposes after the approval of the local ethics committee, Faculty of Medicine, Alexandria, Egypt.

## Consent to participate

Informed written consent was obtained from all patients included in this study.

## Consent to publish

All patients included in this study agreed to the use of their clinical information for scientific publication.

## Authors contributions

All authors contributed to the study conceptualization and design. Material preparation, data collection and analysis were performed by Dr. Abdullah Hammad, Dr. Ghada Abu-Sheasha and Mr Ramy Rashed. The first draft of the manuscript was written by Mr. Ramy Rashed and Mr. Ahmed El-Bakoury, revised and edited by Mr. Ahmed El-Bakoury. All authors commented on previous versions of the manuscript and all authors read and approved the final manuscript.

## Availability of data and material

All the data and material for this study are available at request.
